# Targeting the microRNA-21/AP1 axis by 5-fluorouracil and pirarubicin in human hepatocellular carcinoma

**DOI:** 10.18632/oncotarget.2955

**Published:** 2014-12-10

**Authors:** Xiaodong He, Jingjing Li, Weidong Guo, Wei Liu, Jia Yu, Wei Song, Lei Dong, Fang Wang, Shuangni Yu, Yi Zheng, Songsen Chen, Yan Kong, Changzheng Liu

**Affiliations:** ^1^ Department of General Surgery, Peking Union Medical College Hospital, Chinese Academy of Medical Sciences, Peking Union Medical College, Beijing, PR China; ^2^ Department of Hepatobiliary Surgery, The Affiliated Hospital of Medical College, Qingdao University, Qingdao, PR China; ^3^ Department of Biochemistry and Molecular Biology, State Key Laboratory of Medical Molecular Biology, Institute of Basic Medical Sciences, Chinese Academy of Medical Sciences, School of Basic Medicine Peking Union Medical College, Beijing, PR China; ^4^ Department of Pathology, Peking Union Medical College Hospital, Chinese Academy of Medical Sciences, Peking Union Medical College, Beijing, PR China; ^5^ Key laboratory of Carcinogenesis and Translational Research (Ministry of Education), Department of Renal Cancer and Melanoma, Peking University Cancer Hospital & Institute, Beijing, China

**Keywords:** microRNA-21, AP-1, hepatocellular carcinoma, chemotherapy, HIAC

## Abstract

MicroRNAs function as oncomiRs and tumor suppressors in diverse cancers. However, the utility of specific microRNAs in predicting the clinical benefit of chemotherapy has not been well-established. Here, we investigated the correlation between microRNA-21 expression and hepatic arterial infusion chemotherapy with 5-fluorouracil and pirarubicin (HAIC) for hepatocellular carcinoma (HCC). We found that HCC patients with low microRNA-21 levels in tumors tended to have a longer time to recurrence and disease-free survival. We demonstrated that microRNA-21 suppression in combination with 5-fluorouracil and pirarubicin treatment inhibited tumor growth in subcutaneous xenograft mice models. Mechanistically, the AP-1 and microRNA-21-mediated axis was verified to be a therapeutic target of cytotoxic drugs and deregulation of this axis led to an enhanced cell growth in HCC. Taken together, our findings demonstrate that microRNA-21 is a chemotherapy responsive microRNA and can serve as a prognostic biomarker for HCC patients undergoing HAIC. Targeting microRNA-21 enhances the effect of chemotherapeutic drugs, thereby suggesting that microRNA-21 suppression in combination with HAIC may be a novel approach for HCC treatment.

## INTRODUCTION

Hepatocellular carcinoma (HCC) is a primary liver cancer, the sixth most common cancer, and the third leading cause of cancer-related mortality worldwide [1-3]. Several effective biomarkers and new imaging techniques have enabled HCC detection at an early stage. However, HCC patients still have a high rate of recurrence due to local invasion and intrahepatic metastasis, even after curative therapy [4]. The prognosis of patients with advanced HCC, where the tumor has spread across the liver or has invaded major vessels, remains extremely poor [4-5]. In these patients, conventional therapies using cytotoxic agents or novel targeted drugs have little clinical impact because of poor efficacy and possible complications [6]. Hepatic arterial infusion chemotherapy (HAIC) has several theoretical advantages such as a higher concentration of drugs delivered directly to the tumor and reduced systemic toxicity [7]. However, an optimal protocol of HAIC has not been established for HCC [8]. Therefore, it is imperative to identify useful biomarkers to predict the HCC clinical response to HAIC; these biomarkers might provide novel avenues of HAIC research and aid the early diagnosis and treatment of this highly malignant tumor.

Increasing knowledge of the tumor biology of HCC has elucidated a few coding and non-coding genes involved in hepatocarcinogenesis [9-12]. Although the fact that some of these deregulated genes have been validated as oncogenes or tumor suppressors, little is known regarding whether they can be potential predictors for HAIC. Previous studies have indicated that microRNA (miRNA) expression profiles or signatures can serve as diagnosis tools and predictors for diverse cancers [13-16], and some of these deregulated miRNAs have been validated as oncomiRs or tumor suppressive miRNAs (ts-miRs) in hepatic tumorigenesis[17-18]. For example, miR-122, a specific miRNA in the liver, is downregulated in HCC tissues and acts as a ts-miR through negatively regulating cyclin G1 [19-20]. Various reports focusing on miR-21 have indicated a crucial role for this oncomiR in hepatocarcinogenesis [21-22] and demonstrate the extensive association of miR-21 with HCC prognosis and therapy [23-24]. Similarly, we have previously revealed that miR-21 functions as an oncomiR through suppressing the expression of PTEN, PDCD4, and RECK [21]. These findings imply that miR-21 is a candidate for HCC prognosis and therapy. However, several questions remain unanswered. For example, the correlation between miR-21 expression and the clinicopathological variables in HCC patients is unknown, and it is also unclear whether deregulated miR-21 expression in HCC is a response to HAIC. Chemotherapeutic drugs exert suppressive effects on HCC progression through regulating miR-21 expression.

Herein, we indentify miR-21 as a potential biomarker for predicting HAIC effects. we further demonstrate that chemotherapeutic drugs have clinical promise for HCC therapy through modulating a critical genetic pathway involving an miR-21-mediated program.

## RESULTS

### miR-21 is a potential predictor of HAIC treatment for HCC patients

**Figure 1:**
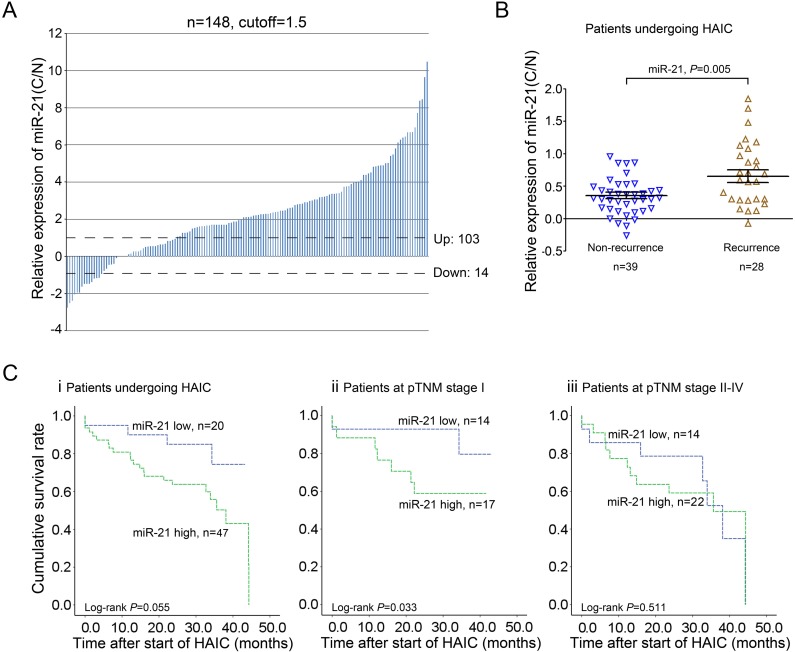
miR-21 is a potential predictor of HAIC treatment with 5-fluorouracil and pirarubicin for HCC patients A: qRT-PCR analysis of miR-21 expression in 148 pairs of HCC tissues (C) and matched adjacent normal liver tissues (N). B: HCC patients with high levels of miR-21 tended to recur after HAIC treatment. C: Kaplan-Meier survival curves for DFS of HCC patients (i), HCC patients at the early stage (ii) and the advanced stage (iii) in relation to miR-21. Cut-off values for miR-21 (high/low expression) were determined through ROC analysis.

Several studies have demonstrated that HAIC has clinical promise for HCC prevention and improves survival [8, 25]. Because miR-21 has been validated as an oncomiR in hepatocarcinogenesis that leads to drug resistance [21, 24, 26], we investigated whether its expression responded to HAIC treatment. In doing so, qRT-PCR analysis was conducted to evaluate miR-21 levels in 148 paired HCC samples, which indicated that miR-21 expression were upregulated in the majority of HCC specimens compared with the matched normal liver tissues (Figure. 1A, Figure. S1A). These data are consistent with our previous findings in 30 paired HCC tissues [21]. In-depth clinicopathological analysis was conducted on 109 patients with detailed information selected from the aforementioned HCC tissues, which showed that high miR-21 levels correlated with a more aggressive tumor phenotype (*P*<0.001) and more extensive invasion (*P*=0.03) (Figure. S1B-C; Supplementary Table 1). The relationship between miR-21 expression and variable pathological grade was also determined, and no significant differences among well, moderate, and poorly differentiated HCC tissues were found (Figure. S1D). A Kaplan-Meier analysis revealed that lower miR-21 levels were associated with longer disease-free survival (DFS) (*P*=0.004; Figure. S1E; Supplementary Table 2). Furthermore, we chose 67 HCC patients undergoing HAIC with 5-fluorouracil and pirarubicin and conducted follow-up studies. These patients were divided into two groups based on recurrence in the 3 years after treatment. We observed that miR-21 expression was substantially higher in the tissues of HCC patients who recurred compared with those without recurrence (Figure. 1B). The Kaplan-Meier method also indicated that HCC patients with low miR-21 levels in tumors tended to have a longer DFS compared with those with high miRNA-21 expression, although the difference did not reach significance (*P*=0.055; Figure. 1C-i). We further conducted Kaplan-Meier analyses on patients with early stage HCC (pTNM stage I) and observed that higher miR-21 levels correlated with shorter DFS in early-stage HCC patients after HAIC (*P*=0.033; Figure. 1C-ii). No significant difference between miR-21-low and miR-21-high groups in HCC patients was found at advanced stages (pTNM stage II, III, and IV; *P*=0.511; Figure. 1C-iii). These findings suggest that HCC patients with lower miR-21 expression levels in tumors have a better response to HAIC treatment, especially at the early stages.

### miR-21 suppression combined with 5-fluorouracil and pirarubicin treatment inhibits HCC xenograft growth

**Figure 2:**
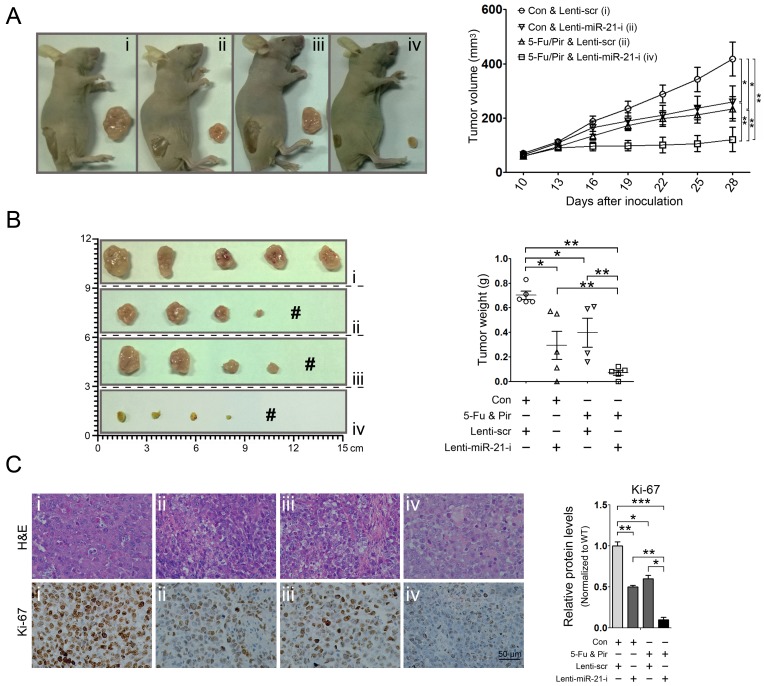
miR-21 suppression combined with 5-fluorouracil and pirarubicin treatment inhibits HCC xenograft growth A and B: miR-21 inhibition in combination with 5-fluorouracil and pirarubicin treatment suppressed HCC xenograft growth. Representative photographs of nude mice (A, left panel) and photographs of dissected tumors from nude mice are shown (B, left panel). # indicates no tumor formation. 5 mice in each group. Graphic representing the tumor volumes at the indicated days (A, right panel). Tumor weight was calculated at the end of the experiment (B, right panel). C: Pathology analysis of tissue sections from recipient mice at 4 weeks post-inoculation. H&E staining and labeling with anti-Ki-67 was performed. Bars: 50 μm.

The above findings promoted us to further investigate whether 5-fluorouracil and pirarubicin treatment represented a better suppressive effect on tumor growth of HCC cells with miR-21 inhibition *in vivo*. To this aim, we conducted miR-21 suppression in Hep3b cells by using a lentiviral system (Lenti-miR-21-i) (Figure. S2A-C) and the subcutaneous xenografts were generated through inoculating the Hep3b cells infected with Lenti-miR-21-i and Lenti-scr, respectively. When the tumor reached a volume of about 100 mm^3^, chemotherapy was started and the tumor size and body weight were determined twice a week during treatment. We observed that miR-21 inhibition combined with 5-fluorouracil and pirarubicin treatment exerted a dramatically suppressive effect on the tumorigenicity of Hep3b cells *in vivo*, as compared to all other experimental groups (Figure. 2A-B; Figure. S2D). Moreover, we performed immunohistochemistry to detect the expression of Ki-67 in randomly selected xenograft mouse tumors, which demonstrated that the above treatment retarded HCC cell growth (Figure. 2C). These results supported our findings in HCC patients who receiving HIAC prevention with 5-fluorouracil and pirarubicin.

### miR-21 expression is suppressed by 5-fluorouracil and pirarubicin in HCC cells

**Figure 3:**
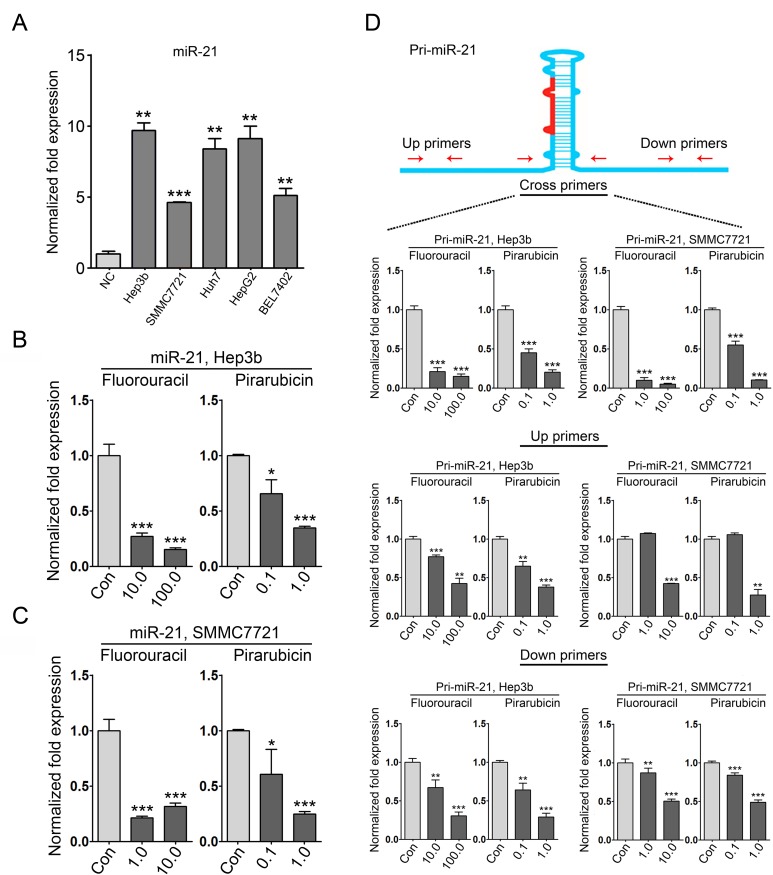
miR-21 expression is suppressed in HCC cells with 5-fluorouracil and pirarubicin treatment A: qRT-PCR analysis was conducted to quantify endogenous miR-21 levels in normal liver (NC), Hep3b, SMMC7721, Huh7, HepG2, and Bel7402 cells. U6 was included as a control, and the data were normalized to the levels of NC. B and C: qRT-PCR analysis was conducted to quantify the expression of miR-21 in Hep3b (B) and SMMC7721 (C) cells following 5-fluorouracil and pirarubicin treatment. U6 was included as a control, and the data were normalized to control treated cells. D: Three pairs of primers were used in the qRT-PCR analysis to evaluate the expression of pri-miR-21 in Hep3b and SMMC7721 cells following 5-fluorouracil or pirarubicin treatment. GAPDH was included as a control, and the data were normalized to control cells.

Based on the fact that HCC patients with low miR-21 levels in tumors had a better response to HAIC treatment with 5-fluorouracil and pirarubicin, we asked whether these two drugs offered a clinical benefit *via* down-regulating miR-21 expression. To this aim, we first evaluated the expression of miR-21 in several HCC cell lines using qRT-PCR (Figure. 3A), and the IC50 values of these drugs in Hep3b (miR-21-high) and SMMC7721 (miR-21-low) cells were determined. The estimated IC50 values for 5-fluorouracil and pirarubicin in the tested cell lines were as follows: Hep3b, 5-fluorouracil, 267.96 mg/L; and SMMC7721, 5-fluorouracil, 131.38 mg/L; Hep3b, pirarubicin, 1.65 mg/L; SMMC7721, pirarubicin, 0.25 mg/L (Figure. S3A-B). Next, Hep3b and SMMC7721 cells were treated with 5-fluorouracil and pirarubicin, and qRT-PCR analyses were performed. We observed that drugs treatment led to a significant decrease in miR-21 levels in the tested HCC cells compared with those treated with physiological saline (control) (Figure. 3B-C). We further investigated whether 5-fluorouracil and pirarubicin exerted their suppressive effect on miR-21 expression at the transcriptional level. qRT-PCR analyses were performed to evaluate the level of primary miR-21 (pri-miR-21) using three paired primers (up, cross, and down primers), which indicated a significant decrease in pri-miR-21 expression in 5-fluorouracil and pirarubicin treated Hep3b and SMMC7721 cells (Figure. 3D).

### 5-fluorouracil and pirarubicin treatment lead to miR-21 downregulation through AP-1 proteins

**Figure 4:**
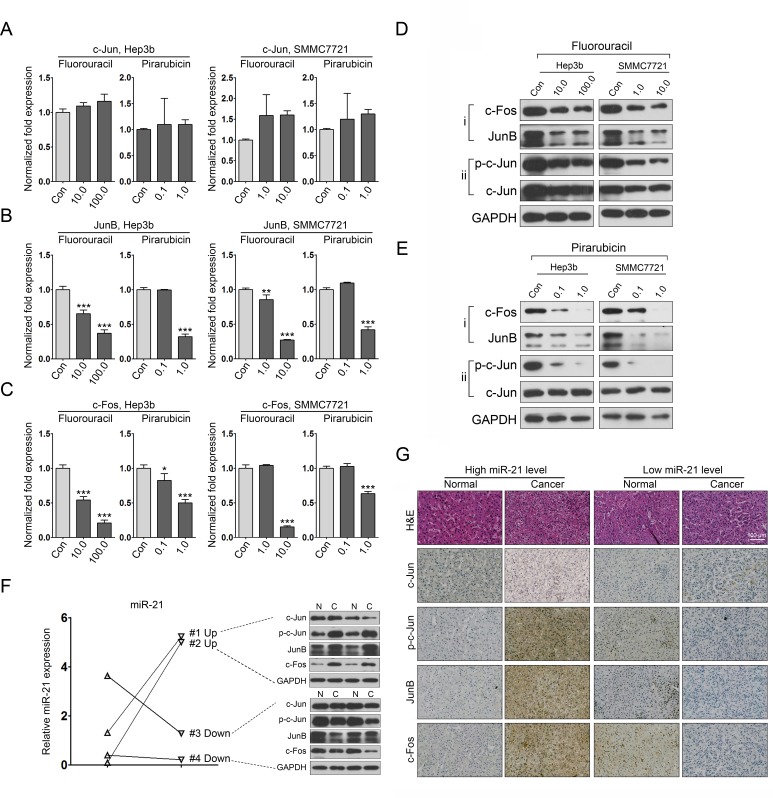
5-fluorouracil and pirarubicin treatment leads to miR-21 downregulation in HCC cells via inhibiting the expression of AP-1 proteins A, B, and C: qRT-PCR analysis was conducted to quantify the expression of AP-1 proteins (c-Jun, A; JunB, B; c-Fos, C) in Hep3b and SMMC7721 cells following 5-fluorouracil or pirarubicin treatment. GAPDH was included as a control, and the data were normalized to control cells. D and E: The protein level of AP-1 components in Hep3b and SMMC7721 cells after treatment with 5-fluorouracil (D) or pirarubicin (E) as measured through immunoblot analysis. GAPDH served as a loading control. F: qRT-PCR analysis of miR-21 expression in 4 pairs of HCC samples. U6 snRNA was used as a loading control. Immunoblot analysis of AP-1 proteins was performed using the same samples. G: Representative images of AP-1 proteins staining in the same samples described in (F). Magnification is 20×. Bars: 100 μm.

Previous studies have demonstrated that the AP-1 transcription factor family is involved in regulating miR-21 expression [27-28]. Thus, we investigated whether the expression of AP-1 components was affected by 5-fluorouracil and pirarubicin. First, we tested the mRNA levels of c-Jun, JunB, and c-Fos using qRT-PCR analyses. Compared with control-treated cells, we observed that the expression of JunB and c-Fos was downregulated in 5-fluorouracil- and pirarubicin-treated HCC cells, and no obvious alteration in c-Jun mRNA levels was noticed (Figure. 4A-C). Further immunoblot analyses demonstrated similar results for the protein levels of JunB and c-Fos (Figure. 4D-i). Interestingly, we observed a marked decrease of p-c-Jun in HCC cells treated with 5-fluorouracil, but no significant change was found in c-Jun protein levels (Figure. 4D-ii). Similar results were found in pirarubicin treated HCC cells (Figure. 4E). Moreover, we evaluated the correlation between AP-1 components and miR-21 in HCC specimens. To this aim, 4 HCC specimens (miR-21-high, 2; miR-21-low, 2) were selected to determine the expression of miR-21 and AP-1 proteins. The analysis revealed that the HCC tissues with augmented AP-1 levels had increased miR-21 expression (Figure. 4F; Figure. S4A). Further immunohistochemical analysis was performed in the same tissue pairs, and AP-1 staining was also increased in HCC tissues with high miR-21 levels compared with the matched normal liver tissues (Figure. 4G; Figure. S4B).

Altogether, these data provide the evidence that 5-fluorouracil and pirarubicin have clinical benefits through modulating the AP-1/miR-21 axis in HCC.

### 5-fluorouracil and pirarubicin suppress HCC cell growth via regulating the miR-21-mediated program

**Figure 5:**
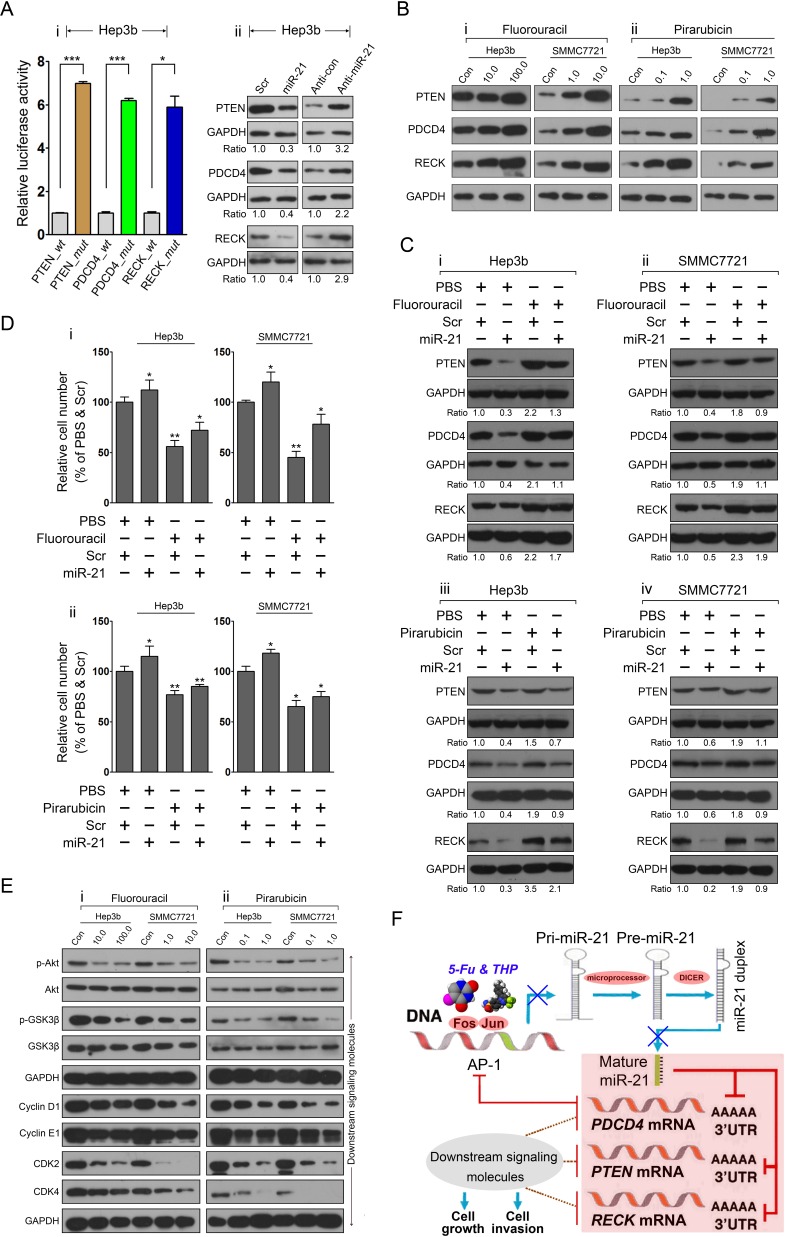
The miR-21-mediated program is modified by 5-fluorouracil and pirarubicin treatment in HCC cells A: The correlation between miR-21 and its targets was validated in Hep3b cells *via* luciferase reporter assay (i) and immunoblots (ii) analysis. GAPDH was included as a loading control, and the data were normalized to scr or anti-control treated cells. The numbers below the panels represent the normalized protein expression levels. B: The protein levels of miR-21 target proteins in Hep3b and SMMC7721 cells after treatment with 5-fluorouracil (i) or pirarubicin (ii) as measured through immunoblot analysis. GAPDH served as a loading control. C: Modulation of miR-21 targets expression in 5-fluorouracil-treated Hep3b (i), SMMC7721 (ii) cells, and pirarubicin-treated Hep3b (iii), SMMC7721 (iv) cells was performed by transfection with miR-21 mimic. The target proteins level was measured through immunoblot analysis. GAPDH served as a loading control. The numbers below the panels represent the normalized protein expression levels. D: HCC cell growth was determined using CCK-8 at 36 h on different treatment conditions as described as (C). E: Immunoblot analyses of p-AKT, AKT, p-GSK-3β, GSK-3β, CDK2, and CDK4 were performed using Hep3b and SMMC7721 cells with 5-fluorouracil (i) and pirarubicin (ii) treatment. GAPDH served as the loading control. F: 5-fluorouracil and pirarubicin have clinical benefit for HCC treatment through modulating the AP-1 and miR-21-mediated axis.

Because an miR-21-mediated PTEN/PDCD4/RECK program has been identified in HepG2 and SMMC7721 HCC cells, we next investigated whether this program was modified by 5-fluorouracil and pirarubicin treatment. The correlation between miR-21 and their targets was further validated in Hep3b cells, indicating that PTEN, PDCD4, and RECK were direct effectors of miR-21 in this cell line (Figure. 5A). We next conducted immunoblot analyses to evaluate the protein levels of PTEN, PDCD4, and RECK in Hep3b and SMMC7721 cells after 5-fluorouracil and pirarubicin treatment and we showed that the tested drugs increased the levels of these proteins, as compared to control-treated cells (Figure. 5B). To further probe the correlation between cell phenotypic alterations and drugs-mediated AP-1/miR-21 axis, we performed a rescue assay that increased and then decreased the level of miR-21 targets *via* drugs treatment in combination with miR-21 overexpression in HCC cells. Immunoblotting was used to evaluate PTEN, PDCD4, and RECK expression and demonstrated that the level of these proteins was altered under different treatment conditions in Hep3b and SMMC7721 cells (Figure. 5C). Furthermore, cell growth analysis was conducted to determine the alterations in cell viability corresponding with the expression level variations of targeted proteins, and we observed that miR-21 overexpression prevent the suppressive impact of 5-fluorouracil (Figure. 5D-i) and pirarubicin (Figure. 5D-ii) treatment on HCC cell growth.

To further characterize the related genetic pathway affected by 5-fluorouracil and pirarubicin involving the miR-21-mediated program in HCC cells, we examined the associated target genes including p-AKT, AKT, p-GSK3β, GSK3β, cyclin D1, cyclin E1, CDK2, and CDK4. Immunoblots were used to analyze the protein expression of these genes in Hep3b and SMMC7721 cells following 5-fluorouracil and pirarubicin treatment. A decrease in p-AKT and p-GSK3β was noted in HCC cells treated with 5-fluorouracil compared with control-treated cells. However, no significant change was observed in total AKT and GSK3β levels. We also observed that cyclin D1, cyclin E1, CDK2, and CDK4 protein levels were decreased in 5-fluorouracil-treated HCC cells (Figure. 5E-i). Similar results were noted in HCC cells with pirarubicin treatment (Figure. 5E-ii).

Collectively, these findings indicate that chemotherapy has a clinical benefit against HCC through modifying the AP-1/miR-21-mediated axis (Figure. 5F).

### Drug-modulating oncomiRs or ts-miRs represent crucial mechanisms for HCC chemotherapy

**Figure 6:**
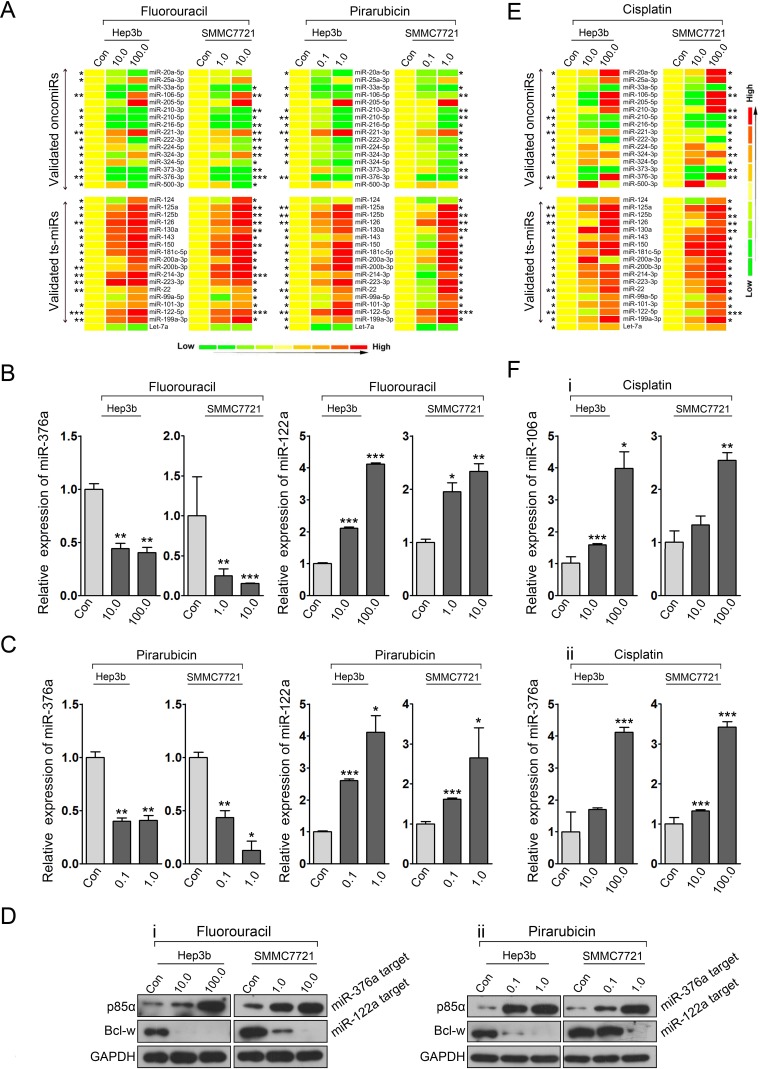
Drug-modulating oncomiRs or ts-miRs represent crucial mechanisms for HCC chemotherapy A: qRT-PCR analysis quantified the expression of candidate oncomiRs and ts-miRs in Hep3b and SMMC7721 cells treated with 5-fluorouracil and pirarubicin. B and C: qRT-PCR analysis was performed to assess the expression of miR-376a and miR-122a in HCC cells treated with 5-fluorouracil (B) and pirarubicin (C). U6 snRNA served as loading controls. D: Immunoblot analysis was conducted to determine the expression of p85α and Bcl-W in HCC cells treated with 5-fluorouracil (i) and pirarubicin (ii). GAPDH served as loading controls. E: qRT-PCR analysis quantified the expression of candidate oncomiRs and ts-miRs in Hep3b and SMMC7721 cells treated with cisplatin. F: qRT-PCR analysis was performed to assess the expression of miR-106a (i) and miR-376a (ii) in HCC cells treated with cisplatin. U6 snRNA served as loading controls.

The above findings prompted us to further investigate the effect of 5-fluorouracil and pirarubicin on other deregulated miRNAs validated in HCC. Hep3b and SMMC7721 cells were treated with these two drugs, and qRT-PCR was performed to measure the expression of selected oncomiRs and ts-miRs. After 5-fluorouracil and pirarubicin treatment, most ts-miRs were upregulated in HCC cells (17/18), and approximately 50% of the test oncomiRs were downregulated (8/16; Figure. 6A and Figure. S5). Importantly, miR-376a-P85α and miR-122a-Bcl-w[29-30], which have been validated as deregulated axes in HCC cells, were modified after 5-fluorouracil and pirarubicin treatment, as shown by using qRT-PCR (Figure. 6B-C) and immunoblot analyses (Figure. 6D). These data suggested that miRNA-mediated programs modified by cytotoxic drugs may represent a global mechanism for HCC chemotherapy activity. In addition, the expression levels of several oncomiRs such as miR-205 and miR-221 were significantly induced by 5-fluorouracil and pirarubicin treatment (Figure. S6), which might lead to chemoresistance in HCC.

Taken together, our findings suggest that multiple carcinogenesis-related programs, including oncomiR- or ts-miR-mediated axes, are involved in the HCC response to chemotherapy.

## DISCUSSION

Increasing evidence clearly demonstrate that miR-21 not only is a crucial tumor promoter in hepatic tumorigenesis but also functions as a prognostic factor in HCC patients[21-22, 31]. Recent studies have also revealed the prognostic value of circulating miRNAs for detecting cancer[32]. For example, circulating miR-21 serves as a biomarker for HCC diagnosis [23, 33]. However, the link between deregulated miR-21 and the prognosis of HCC patients undergoing HAIC has not been established. Here, we indicated that patients with higher miR-21 expression in tumors tended to relapse, which implied the prognostic significance of miR-21 for predicting the clinical benefit of HAIC with 5-fluorouracil and pirarubicin for HCC treatment. We also showed that HCC patients with a longer DFS tended to express lower miR-21 levels in tumors, albeit not to a significant degree (*P*=0.055). The different clinical stages of the patients may contribute to this finding. Thus, HCC patients were divided into 2 groups, namely, early stage (pTNM I) and advanced stage (pTNM II-IV), based on our correlation analysis between miR-21 expression and pTNM stage. We observed that HCC patients at an early stage with lower miR-21 expression levels had a longer DFS after 5-fluorouracil and pirarubicin treatment via HAIC. We further generated subcutaneous xenografts by inoculating Hep3b cells with or without miR-21 inhibition to evaluate the antitumor effect of 5-fluorouracil and pirarubicin treatment *in vivo*, which indicated that miR-21 suppression in combination with 5-fluorouracil and pirarubicin treatment markedly inhibited HCC xenograft growth.

AP-1 activation plays a major role in tumor promotion and progression *via* regulating the expression of several oncogenes, which suggest that this transcriptional factors family might serve as a potential target for tumor therapy[34]. Several small molecular extracted from plant offer chemopreventive effects on tumorigenesis through inhibiting AP-1 activation, supporting this idea[35]. However, AP-1 is composed of a mixture of homo- and heterodimers of Jun and Fos proteins, which results in a complex phenotypic alteration of cells via regulating different target genes[36]. For example, ectopic expression of GADD153, which is also a putative target gene of AP-1, increases the sensitivity of MKN45 gastric cancer cells to VP-16, cisplatin, 5-fluorouracil, and docetaxel, indicating that augmented GADD153 expression and AP-1 binding activity promote apoptosis induced by anticancer drugs[37]. In the present study, we demonstrated that 5-fluorouracil and pirarubicin offered chemopreventive effects *via* inhibiting AP-1 activation, thereby leading to a modification of miR-21-mediated program in HCC cells. Our data suggest that the AP-1 pathway is a good target not only for the prevention but also for the therapy of HCC.

Cisplatin is another cytotoxic drugs widely used in HAIC for HCC treatment. We also investigated whether cisplatin exerted its suppressive effects on HCC cells via inhibiting miR-21 expression. Although several reports indicate that miR-21 inhibition lead to an improvement of cisplatin sensitivity[38-39], our qRT-PCR analysis revealed that cisplatin treated-Hep3b and SMMC7721 cells depicted no significant change in miR-21 levels (Figure. S7). These data suggest that the affected genetic pathways of cisplatin might be different with 5-fluorouracil and pirarubicin. We further conducted cisplatin treatment in HCC cells and performed expression analyses of some oncomiRs and ts-miRs tested in 5-fluorouracil and pirarubicin treatment. A different miRNA expression profile was observed in cisplatin treated cells compared with 5-fluorouracil and pirarubicin treatment (Figure. 6E-F). These findings represent a global mechanism for chemotherapy achieving clinical promise for HCC prevention through regulating miRNA-mediated axes but drugs may offer their effects through different genetic pathways.

Taken together, our findings indicate that HAIC prevention using 5-fluorouracil and pirarubicin has clinical promise for HCC patients through modulating the AP-1 and miR-21 axis. miR-21 could function as a potential biomarker for predicting the prognosis of HCC patients receiving HAIC treatment.

## MATERIALS AND METHODS

### Human Liver Tissues and Cell Lines

This study was approved by the ethical board of the Peking Union Medical College hospital and the ethical board of the Institute of Basic Medical Sciences, Chinese Academy of Medical Sciences, and all samples were obtained with patients' informed consent. Paired samples of tumor/non-tumor liver tissues were collected from patients undergoing HCC surgery, immediately snap frozen in liquid nitrogen, and stored at −80°C until RNA extraction. The patient characteristics are provided in Supplementary Table 1. Follow-up studies were performed on 67 HCC patients who underwent HAIC. The total RNA pool of normal liver samples comprised RNA from at least 3 donors, and the information for each healthy donor, including the age, sex, race, cause of death, and diagnosis, was obtained from Ambion Inc.

HCC cell lines, Hep3b, and SMMC7721 were purchased from the Shanghai Cell Bank, Chinese Academy of Sciences and cultured under standard conditions.

### RNA isolation and quantitative real-time PCR (qRT-PCR) analysis

Total RNA was extracted from cells and tissues using Trizol (Invitrogen, Carlsbad, CA, USA) according to the manufacturer's instruction. The RNA was quantified by absorbance at 260 nm. To assess the levels of miR-21, qRT-PCR analysis was conducted by using Taqman probes (Invitrogen, Carlsbad, CA, USA) in the Bio-Rad IQ5 qRT-PCR system according to the manufacturer's instruction. The data were normalized using endogenous U6 snRNA. The expression levels of miRNAs in cancer relative to its non-tumorous control and HCC cells were calculated using the equation 2^−ΔΔCT^ in which ΔC_T_=C_T_ 21-C_T_ U6. The value of the relative expression ratio <1.0 was considered as low expression in cancer relative to the non-tumorous control, where others were considered as high expression. To assess the levels of pri-miR-21 and AP-1 components in tested HCC cells, qRT-PCR analysis was conducted by using Taqman probes (Invitrogen, Carlsbad, CA, USA) in the Bio-Rad IQ5 qRT-PCR system according to the manufacturer's instruction. The data were normalized using endogenous GAPDH. Primers for qRT-PCR are shown in Supplementary Table 3.

### Constructs, reagents and assays

The perfect complementary sequence of miR-21 and the 3′UTRs of the human PTEN, PDCD4, and RECK mRNA were cloned in between the Not1 and *Xba*1 sites of pRL-TK (Promega). Sequences of the other primers were shown in Supplementary Table 3. MiRNA mimics specifying miR-21 and control miRNA mimic were obtained from Dharmcon Inc. Cisplatin is a product of Hospira Austrila Pty Ltd., 5-fluorouracil is a product of Tianjin Kingyork Amino Acid Co., Ltd., and pirarubicin is a product of Shenzhen Main Luck Pharmaceuticals Inc.

Hep3b cells were seeded onto 24-well plates (1×10^5^ cells per well) the day before transfections. Cells (≈70% confluent) were transfected with pRL-TK luciferase reports (50 ng per well), pGL3-control firefly luciferase (10 ng per well). All transfections were carried out in triplicate with Effectene (QIAGEN). Cell lysates were prepared with Passive Lysis Buffer (Promega) 48 h after transfection, and luciferase activities were measured by using the Dual Luciferase Reporter Assay (Promega).

### Cell growth assay

The cellular proliferation rate was measured using CCK-8 (DOJINDO) as previously described[21]. To measure the effects on cellular proliferation rates, cells were incubated in 10% CCK-8 (DOJINDO) diluted in normal culture media at 37 °C until the appearance of visual color. Proliferation rates were determined at 12, 24, 36, 48, and 60 h post-transfection and quantification was done on a microtiter plate reader (Spectra Rainbow, Tecan) using the protocol recommended by the manufacturer.

### Western blots

We extracted proteins from Hep3b, SMMC7721 cells, and HCC tissues with mammalian cell lysis buffer M-PER (PIERCE) containing protease and phosphatase inhibitor. Proteins from total cell lysates were resolved with a 4-20% Tris-HCl gradient gel (Bio-Rad), transferred to PVDF membranes, blocked in 5% nonfat milk or BSA in TBS/Tween-20, and blotted with antibodies for PTEN, PDCD4, RECK, c-Jun, JunB, c-Fos, p-AKT, AKT, p-GSK3β, GSK3β, cyclin D1, cyclin E1, CDK2, CDK4, and GAPDH (RECK and c-Jun from BD biosciences, cyclin D1, cyclin E1, CDK2, CDK4, and GAPDH from Abcam, others from CST).

### Immunohistochemistry

HCC tissues and mouse tumor tissues were made into paraffin sections and pretreated at 65°C for 2 hours, followed by deparaffinization. Antigen retrieval was carried out before application of the primary antibodies (Ki-67, 1:100; DAKO), overnight at 4°C. As a negative control, sections were incubated with normal IgG. Thereafter, slides were incubated for 2 hours at room temperature with the secondary antibody conjugated to horseradish peroxidase (HRP; 1:100; DAKO). HRP activity was detected using the Liquid DAB+ Substrate Chromogen System (DAKO). Finally, sections were counterstained with hematoxylin and photographed.

### Evaluation of immunohistochemical staining

Immunohistochemistry stained sections were examined using an Olympus Vanox-T AH-2 light microscope (Olympus, Tokyo, Japan). The same magnification (microscope objective) was used to record all images in a particular series. Both the field limiting and contrast apertures were kept at the fully open position during (digital) photography to avoid any variability in reproducing aperture settings. Images were recorded with a Spot RTCCD camera (Diagnostic Instruments, Sterling Heights, MI, USA) in color mode, using full (1600× 1200 bit) resolution at 8-bit depth for each (RGB) color component. The photographs were taken with identical exposure settings. A flat field image was obtained at the beginning of each microscopy session and then used throughout the session to correct for uneven illumination. The white balance was adjusted for each slide. Recorded images were stored in color mode as uncompressed files in tagged image file format. The subsequent digital images were analyzed using the built-in functions of ImagePro Plus image analysis software 6.0 (Media Cybernetics, Silver Spring, MD, USA) by three pathologists simultaneously. Five fields of view were chosen stochastically by each pathologist. The procedure for determining the immunostaining intensity was as previously described[40].

### HCC xenograft model

All animal procedures were performed according to the national Animal Experimentation guidelines (D.L.116/92) upon approval of the experimental protocol by the Institutional Animal Experimentation Committee of Peking Union Medical College. For the xenograft assay, six-week-old female nude mice (BALB/c-nude) were used to examine tumorigenicity. Hep3b HCC cells with or without miR-21 inhibition by using lentiviral system (Lenti-miR-21-I and Lenti-scr) were propagated (6 × 10^6^ cells saline/Matrigel (BD Pharmigen San Jose, Ca), 1:1 v/v) and inoculated s.c. into the dorsal flanks of 20 mice (5 for each group as described). The size of the tumors was measured by caliper twice a week, and tumor volumes were calculated using the following formula: Л/6×d^2^×D removed and weighed 7 weeks after tumor cell injection. Treatments were started after tumor reached approximately 100 mm^3^. 5-fluorouracil (500 mg/m^2^) and pirarubicin (20 mg/m^2^) were administered once a week, intravenously. Control mice received physiological saline only, according to the same schedule.

### Statistics

Each experiment was repeated at least 3 times. Student's t test (2-tailed) was performed to compare 2 groups (*P*<0.05 was considered significant) unless otherwise indicated (X^2^ test), and data for 3 groups were analyzed using 1-way analysis of variance. Correlations between miRNA expression and clinicopathological features were analyzed using non-parametric tests, such as the Mann-Whitney *U*-test for differences between 2 groups and the Kruskal-Wallis test for differences among 3 or more groups. All statistical analyses were performed using SPSS 16.0 software (SPSS Inc., Chicago, IL, USA). *P*-values <0.05 were considered significant.

## SUPPLEMENTARY MATERIAL, FIGURES, TABLES


