# Supplementary material for: Targeting the microRNA-21/AP1 axis by 5-fluorouracil and pirarubicin in human hepatocellular carcinoma
Source: Oncotarget. 2014 Dec 10;6(4):2302–14. doi: 10.18632/oncotarget.2955 (PMC4385853; doi:10.18632/oncotarget.2955)
Supplement: Supplementary file 1 [file oncotarget-06-2302-s001.pdf]

Targeting the microRNA-21/AP1 axis by 5-fluorouracil and pirarubicin in human hepatocellular carcinoma

Supplementary Material

Supplementary table 1: Comparison of miR-21 expression levels with clinic-pathological features in patients with primary human hepatocellular carcinoma.

Clinic-pathological parameters	N	Median expression of miR-21[1]	*P*
**Age, y**			0.6789
≥60	45	2.352 ± 0.2918	
<60	64	2.543 ± 0.3569	
**Gender**			0.3575
Male	91	2.524 ± 0.2461	
Female	18	1.963 ± 0.5595	
**Tumor Numbers**			0.120
**1**	68	2.160 ± 0.2989	
**≥2**	41	2.881 ± 0.3277	
**Degree of differentiation**			0.4661
Well	34	2.462 ± 0.4844	
Moderately	33	2.195 ± 0.3911	
Poorly	42	2.929 ± 0.4140	
**T Classification**			0.0069 *
T1 + T2	76	2.034 ± 0.2682	
T3 + T4	43	3.345 ± 0.3730	

**N Classification**			0.0767
**N0**	103	2.355 ± 0.2302	
**N1**	6	4.082 ± 0.8670	
**M Classification**			0.0054 *
**M0**	91	2.155 ± 0.2402	
**M1**	18	3.825 ± 0.5179	
**TNM stage**			<0.001 *
I	50	1.441 ± 0.2582	
II	15	2.374 ± 0.7138	
III	24	3.057 ± 0.3788	
IV	20	4.217 ± 0.5728	
**Extrahepatic Spread**			0.0284 *
Negative	64	2.019 ± 0.2556	
Positive	45	3.017 ± 0.3936	
**HBV infection**			0.9418
Negative	27	2.402 ± 0.4342	
Positive	82	2.440 ± 0.2643	
**Liver cirrhosis**			0.0284 *
Negative	64	2.019 ± 0.2556	
Positive	45	3.017 ± 0.3936	
**Liver ascites**			0.5311
Negative	83	2.510 ± 0.2548	

Positive	26	2.178 ± 0.4844	
**AFP levels**			0.2840
Normal	45	2.719 ± 0.3500	
Abnormal	64	2.228 ± 0.2936	
**ALT levels**			0.9126
Normal	57	2.455 ± 0.3550	
Abnormal	52	2.405 ± 0.2706	

1. Mean ± SD, mean of $Log_{1.5}$ (C/N), Standard Definition of $Log_{1.5}$ (C/N). C, normalized expression of cancer tissues; N, normalized expression of adjacent noncancerous tissues.

2. * Indicated statistical significance (P<0.05).

Supplementary table 2: miR-21 associated with disease-free survival of patients with HCC in the training set

No.	miRNA	›ld change (C/N)	LOG (C/N)	miR-21 high (n)	miR-21 low (n)	HR (95% CI)	*P*[a]
1	miR-21	1.90	1.583006	74	35	67.99%-84.18%	<0.0001
2[b]	miR-21	1.87	1.543754	47	20	48.88%-68.06%	<0.0001
3[b]	miR-21	1.83	1.490427	17	14	72.52%-91.51%	0.0041
4[b]	miR-21	2.36	2.11772	22	14	77.53%-98.25%	<0.0001

3. [a] Cox proportional hazard regression analysis; [b] These patients received HAIC prevention.

Supplementary table 3: Sequences of miR-21, OncomiRs, Ts-miRs, and AP-1 family primers.

Target ID		Sequence
hsa-miR-21 precursor		AUGACUGAUUUCUUUUGGUGUUCAGAGUCAAUAUAAUUUUCUAGCACCAUCUGA AAUCGGUUAU
hsa-miR-21 mature sequence		UAGCACCAUCUGAAAUCGGUUA
miR-21 RT		5’ GTCGTATCCAGTGCAGGGTCCGAGGTATTCGCACTGGATACGACTAACCG 3’
miR-21	Forward	5’ TGCGCTAGCACATCTGAAAT 3’
miRNA	Reverse	5’ GTGCAGGGTCCGAGGT 3’
U6 snRNA RT		5’AAAATATGGAACGCTTCACGAATTTG 3’
U6 snRNA	Forward	5’ CTCGCTTCGGCAGCACATATACT 3’
	Reverse	5’ ACGCTTCACGAATTTGCGTGTC 3’
Let-7a-5p RT		5’ GTCGTATCCAGTGCAGGGTCCGAGGTATTCGCACTGGATACGACAACTAT 3’
miR-101-3p RT		5’ GTCGTATCCAGTGCAGGGTCCGAGGTATTCGCACTGGATACGACAACTAT 3’
miR-106b-5p RT		5’ GTCGTATCCAGTGCAGGGTCCGAGGTATTCGCACTGGATACGACATCTGCA 3’
miR-122-5p RT		5’ GTCGTATCCAGTGCAGGGTCCGAGGTATTCGCACTGGATACGACAACTAT 3’
miR-124-3p RT		5’ GTCGTATCCAGTGCAGGGTCCGAGGTATTCGCACTGGATACGACGGCATT 3’
miR-125a-5p RT		5’ GTCGTATCCAGTGCAGGGTCCGAGGTATTCGCACTGGATACGACTCACAGG 3’
miR-125b RT		5’ GTCGTATCCAGTGCAGGGTCCGAGGTATTCGCACTGGATACGACTAACCGA 3’
miR-126-3p RT		5’ GTCGTATCCAGTGCAGGGTCCGAGGTATTCGCACTGGATACGACCGCATTA 3’
miR-130a-3p RT		5’ GTCGTATCCAGTGCAGGGTCCGAGGTATTCGCACTGGATACGACATGCCCT 3’
miR-143-3p RT		5’ GTCGTATCCAGTGCAGGGTCCGAGGTATTCGCACTGGATACGACGAGCTA 3’

miR-150 RT	5’ GTCGTATCCAGTGCAGGGTCCGAGGTATTCGCACTGGATACGACAACTAT 3’
miR-181c-5p RT	5’ GTCGTATCCAGTGCAGGGTCCGAGGTATTCGCACTGGATACGACACTCACC 3’
miR-199a-3p RT	5’ GTCGTATCCAGTGCAGGGTCCGAGGTATTCGCACTGGATACGACTAACCAA 3’
miR-20a-5p RT	5’ GTCGTATCCAGTGCAGGGTCCGAGGTATTCGCACTGGATACGACCTACCTG 3’
miR-200a-3p RT	5’ GTCGTATCCAGTGCAGGGTCCGAGGTATTCGCACTGGATACGACACATCGT 3’
miR-200b-3p RT	5’ GTCGTATCCAGTGCAGGGTCCGAGGTATTCGCACTGGATACGACAACTAT 3’
miR-205-5p RT	5’ GTCGTATCCAGTGCAGGGTCCGAGGTATTCGCACTGGATACGACCAGACTC 3’
miR-210-3p RT	5’ GTCGTATCCAGTGCAGGGTCCGAGGTATTCGCACTGGATACGACTCAGCCG 3’
miR-210-5p RT	5’ GTCGTATCCAGTGCAGGGTCCGAGGTATTCGCACTGGATACGACCAGTGTG 3’
miR-214-3p RT	5’ GTCGTATCCAGTGCAGGGTCCGAGGTATTCGCACTGGATACGACACTGCCT 3’
miR-216-5p RT	5’ GTCGTATCCAGTGCAGGGTCCGAGGTATTCGCACTGGATACGACTCACAGT 3’
miR-22-3p RT	5’ GTCGTATCCAGTGCAGGGTCCGAGGTATTCGCACTGGATACGACACAGTTC 3’
miR-221-3p RT	5’ GTCGTATCCAGTGCAGGGTCCGAGGTATTCGCACTGGATACGACTCATACA 3’
miR-222-3p RT	5’ GTCGTATCCAGTGCAGGGTCCGAGGTATTCGCACTGGATACGACACAAAG 3’
miR-223-3p RT	5’ GTCGTATCCAGTGCAGGGTCCGAGGTATTCGCACTGGATACGACTGGGGTA 3’
miR-224-5p RT	5’ GTCGTATCCAGTGCAGGGTCCGAGGTATTCGCACTGGATACGACAACGGAA 3’
miR-25a-3p RT	5’ GTCGTATCCAGTGCAGGGTCCGAGGTATTCGCACTGGATACGACTCAGACC 3’
miR-324-5p RT	5’ GTCGTATCCAGTGCAGGGTCCGAGGTATTCGCACTGGATACGACACACCAA 3’
miR-324-3p RT	5’ GTCGTATCCAGTGCAGGGTCCGAGGTATTCGCACTGGATACGACCCAGCAG 3’
miR-33a-5p RT	5’ GTCGTATCCAGTGCAGGGTCCGAGGTATTCGCACTGGATACGACTGCAATG 3’
miR-373-3p RT	5’ GTCGTATCCAGTGCAGGGTCCGAGGTATTCGCACTGGATACGACGGCATT 3’
miR-376a-3p RT	5’ GTCGTATCCAGTGCAGGGTCCGAGGTATTCGCACTGGATACGACACGTGGA 3’

miR-500a-3p RT		5’ GTCGTATCCAGTGCAGGGTCCGAGGTATTCGCACTGGATACGACCAGAATC 3’
miR-99a-5p RT		5’ GTCGTATCCAGTGCAGGGTCCGAGGTATTCGCACTGGATACGACAACTAT 3’
Let-7a-5p	Forward	5’ TGCGCTGAGGTAGTAGGTTGT 3’
miR-101-3p	Forward	5’ ACTAAATCGGATCCGTCTGAG 3’
miR-106b-5p	Forward	5’ AGCTCTCTAAAGTGCTGACAG 3’
miR-122-5p	Forward	5’ CCTCGTCCAGTTTTCCCAGG 3’
miR-124-3p	Forward	5’ GCTAAGGCACGCGGTG 3’
miR-125a-5p	Forward	5’ GAGGTCCCTGAGACCCTTTAA 3’
miR-125b	Forward	5’ GCTGTTAACTGGCCTACAAAG 3’
miR-126-3p	Forward	5’ AGCTACTCGTACCGTGAGTAA 3’
miR-130a-3p	Forward	5’ GCTGCCCAGTGCAATGTTAAA 3’
miR-143-3p	Forward	5’ GCGCTGAGATGAAGCACTG 3’
miR-150	Forward	5’ CAGCAATGTAGTGTTTCCTACT 3’
miR-181c-5p	Forward	5’ CTGGAGAACATTCAACCTGTC 3’
miR-199a-3p	Forward	5’ AGCTGTACAGTAGTCTGCACA 3’
miR-20a-5p	Forward	5’ GCCCCTAAAGTGCTTATAGTG 3’
miR-200a-3p	Forward	5’ AGCAGCTAACACTGTCTGGTA 3’
miR-200b-3p	Forward	5’ CAGCAATCTTTGGTTATCTAGC 3’
miR-205-5p	Forward	5’ GAGAGTCCTTCATTCCACCG 3’
miR-210-3p	Forward	5’ CTACAACTGTGCGTGTGACAG 3’
miR-210-5p	Forward	5’ ATATAGCCCCTGCCCACCG 3’
miR-214-3p	Forward	5’ GTTTTTACAGCAGGCACAGAC 3’

miR-216-5p	Forward	5’ AGCTAGTAATCTCAGCTGGCA 3’
miR-22-3p	Forward	5’ AGCTCTAAGCTGCCAGTTGAA 3’
miR-221-3p	Forward	5’ AGCATCATGATGGGCTCCT 3’
miR-222-3p	Forward	5’ AGCATCATGATGGGCTCCT 3’
miR-223-3p	Forward	5’ GCAGCCTGTCAGTTTGTCAAA 3’
miR-224-5p	Forward	5’ AGCTACTCAAGTCACTAGTGG 3’
miR-25-3p	Forward	5’ GAGAGACATTGCACTTGTCTC 3’
miR-324-5p	Forward	5’ ATATCGCATCCCCTAGGGCA 3’
miR-324-3p	Forward	5’ AGTATATAACTGCCCCAGGTG 3’
miR-33a-5p	Forward	5’ CACACACGTGCATTGTAGTTG 3’
miR-373-3p	Forward	5’ CAAAATGGGTCTTTGCGGGC 3’
miR-376a-3p	Forward	5’ CTGCTCTCATCATAGAGGAAAA 3’
miR-500a-3p	Forward	5’ ACTATTATGCACCTGGGCAAG 3’
miR-99a-5p	Forward	5’ CACAAAATTTGTTCGTTCGGCT 3’
Pri-miR-21		
Up primer	Forward	5’ GAATTGGGGTTCGATCTTAACAG 3’
	Reverse	5’ ACGATGGTAGGCAAAACAAGC 3’
Cross primer	Forward	5’ ATCCTGCCTGACTGTCTGCT 3’
	Reverse	5’ CGGCAATGATGCTGGGTAATG 3’
Down primer	Forward	5’ TTCATCTGACCATCCATATCCA 3’
	Reverse	5’ TCTCCATAAAATCCTCCCTCC 3’
c-Jun	Forward	5’ TCCAAGTGCCGAAAAAGGAAG 3’

	Reverse	5’ CGAGTTCTGAGCTTTCAAGGT 3’
JunB	Forward	5’ ACGACTCATACACAGCTACGG 3’
	Reverse	5’ GCTCGGTTTCAGGAGTTTGTAGT 3’
c-Fos	Forward	5’ CCGGGGATAGCCTCTCTTACT 3’
	Reverse	5’ CCAGGTCCGTGCAGAAGTC 3’
GAPDH	Forward	5’ GCACCACCAACTGCTAG 3’
	Reverse	5’ GATGCAGGGATGATGTTC 3’

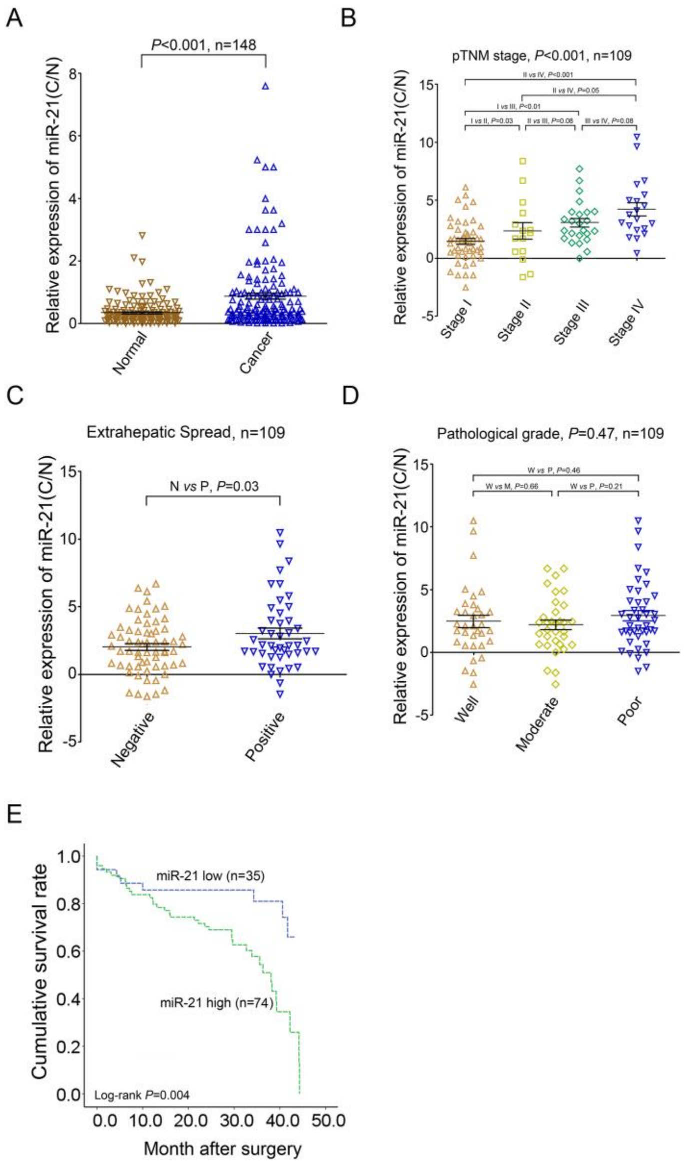

Figure. S1: miR-21 expression is upregulated in HCC tissues. **A:** qRT-PCR analysis is conducted to evaluate the expression of miR-21 in 148 pairs of HCC tissues (Cancer) and the matched adjacent normal liver tissues (Normal) and indicates an increase of miR-21 level in HCC tissues. **B:** Correlation of miR-21 expression with the clinical stage of the HCC patients used for miR-21 expression analysis (n=109). **C:** Correlation of miR-21 expression with the extrahepatic spread of the HCC patients used for miR-21 expression analysis (n=109). **D:** Correlation of miR-21 expression with the pathological grade of the HCC samples used for miR-21 expression analysis (n=109). **E:** Correlation of miR-21 expression with the DFS of the HCC patients (n=109)

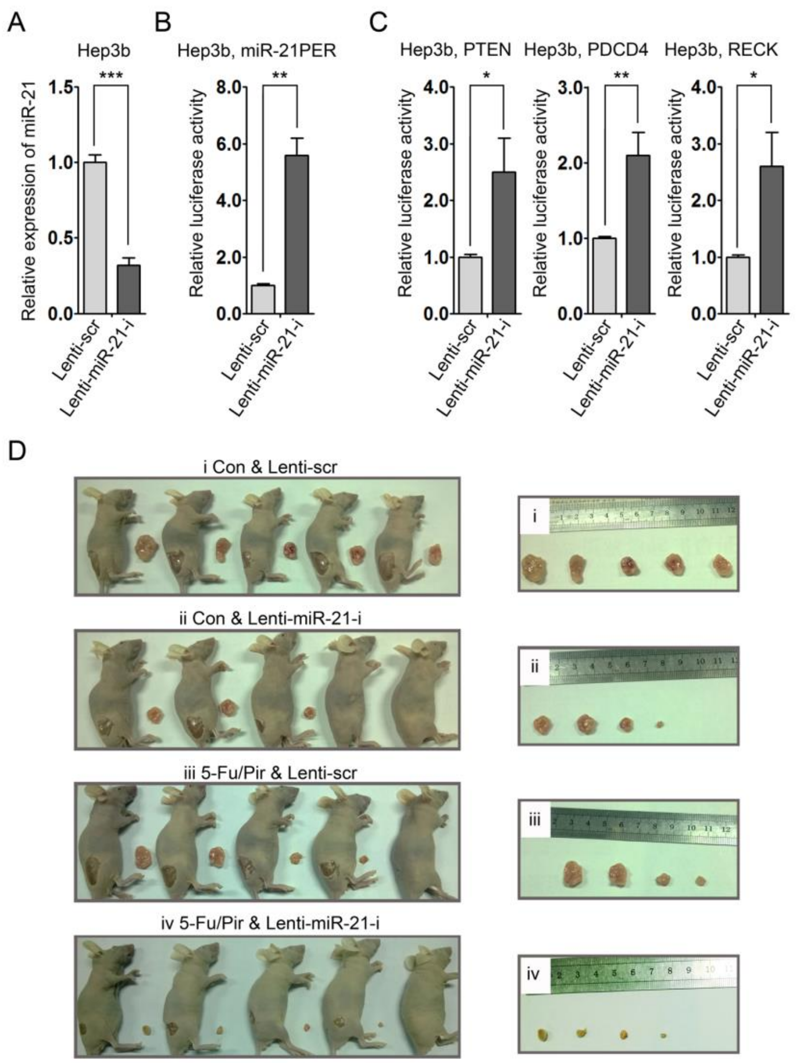

Figure. S2: miR-21 inhibition in combination with 5-fluorouracil and pirarubicin treatment suppress HCC xenograft growth. A: miR-21 expression was downregulated in Hep3b HCC cells infected with Lenti-miR-21-i compared with Lenti-scr-treated cells. **B:** Luciferase assay of Hep3b HCC cells transfected pRL-TK-miR-21 PER following Lenti-miR-21-i or Lenti-scr infection shows that endogenous miR-21 was markedly suppressed. **C:** Luciferase assay of Hep3b HCC cells transfected pRL-TK-PTEN (left panel), pRL-TK-PDCD4 (middle panel), or pRL-TK-RECK (right panel) following Lenti-miR-21-i or Lenti-scr infection shows that endogenous miR-21 was markedly suppressed. **D:** Representative photographs of nude mice (left panel) and photographs of dissected tumors from nude mice are shown (right panel).

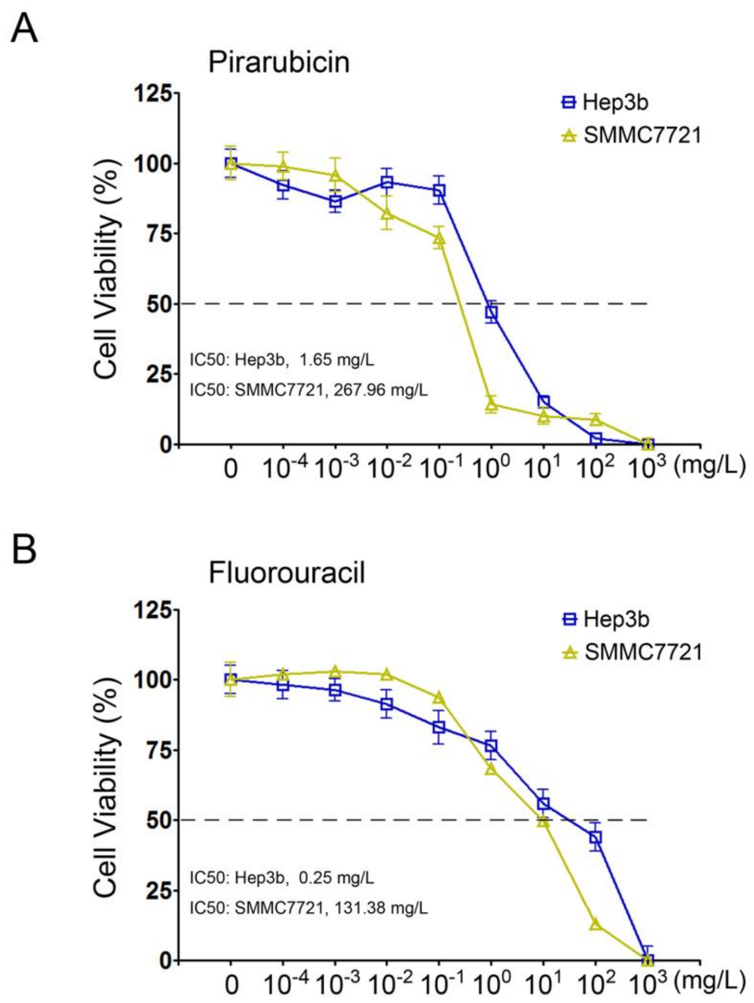

Figure. S3. IC50 values of pirarubicin and 5-fluorouracil is determined in HCC cells.

A-B: IC50 values of pirarubicin (A) and 5-fluorouracil (B) were determined in HCC cells by evaluating cell viability using the CCK-8 assay.

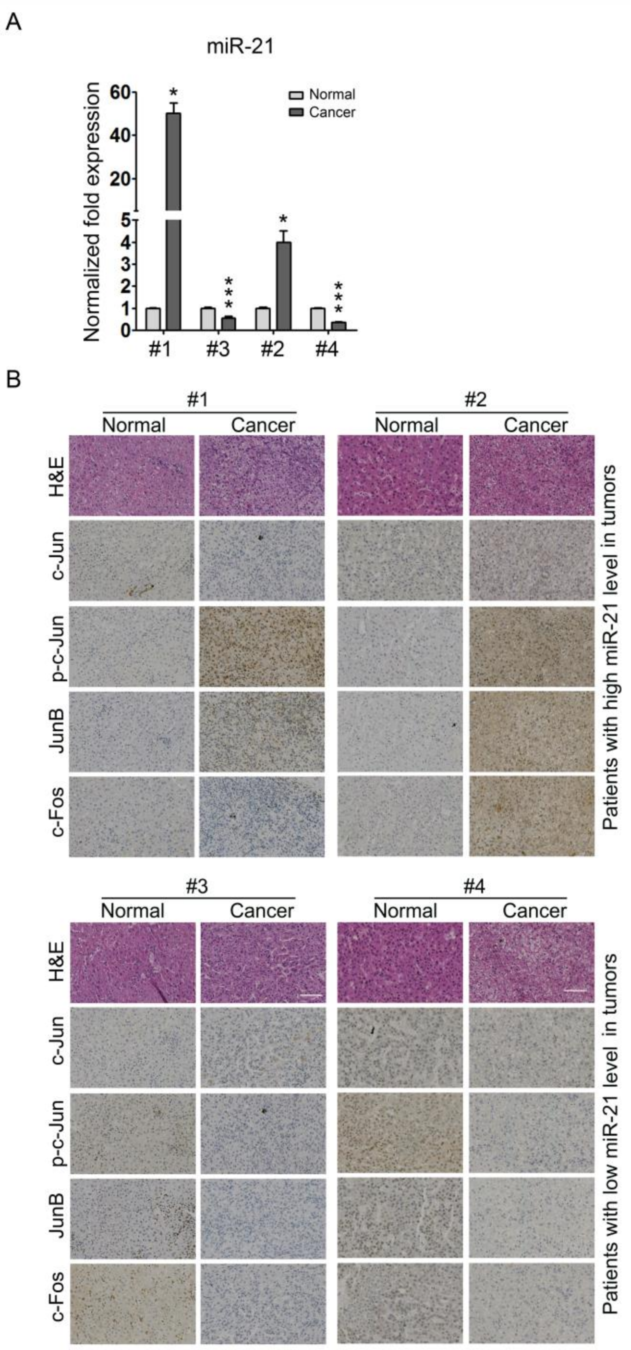

Figure. S4: Expression of miR-21 and AP-1 proteins in HCC tissues. A: qRT-PCR analysis is conducted to evaluate the expression of miR-21 in tissues from four paired of HCC patients. #1 and #2 indicate HCC patients with increasing expression of miR-21 in tumors; #3 and #4 indicate HCC patients with decreasing expression of miR-21 in tumors. Normal indicates the matched normal liver tissues and cancer indicates HCC tissues. **B:** Representative images of AP-1 proteins staining in the same samples described in Figure. S5. Magnification is 20×. Bars: 100 μm.

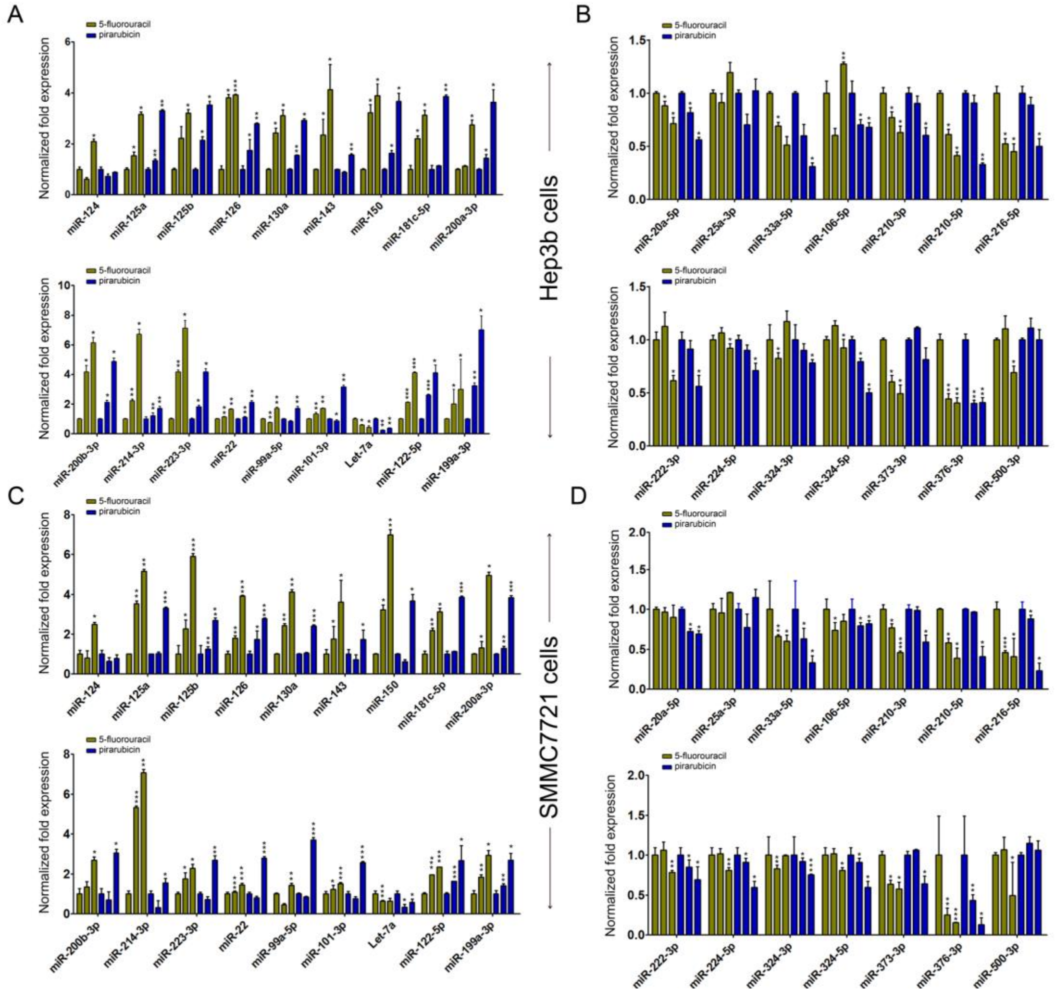

Figure. S5. The expression of candidate oncomiRs and ts-miRs is evaluated in Hep3b and SMMC7721 cells treated with 5-fluorouracil and pirarubicin. A-B, qRT-PCR analysis quantified the expression of candidate oncomiRs and ts-miRs in Hep3b cells treated with 5-fluorouracil and pirarubicin (ts-miRs, A; oncomiRs, B). (C-D) SMMC7721 cells treated with 5-fluorouracil and pirarubicin (ts-miRs, C; oncomiRs, D).

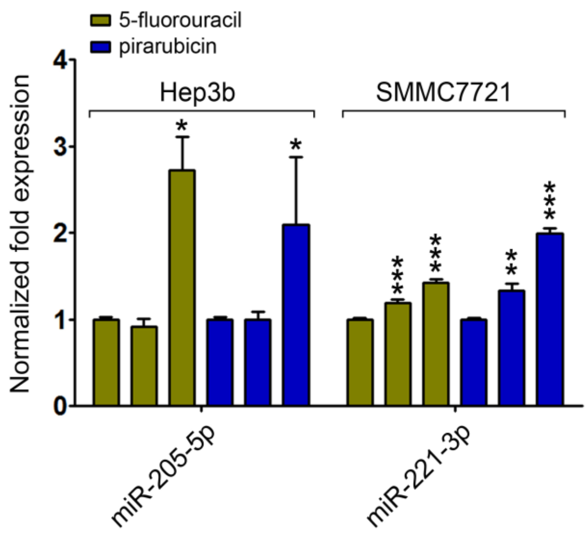

Figure. S6: The expression of miR-205-5p and miR-221-3p is evaluated in HCC cells upon chemotherapeutic drugs treatment. qRT-PCR analysis quantified the expression of miR-205-5p and miR-221-3p in Hep3b and SMMC7721 cells treated with 5-fluorouracil and pirarubicin.

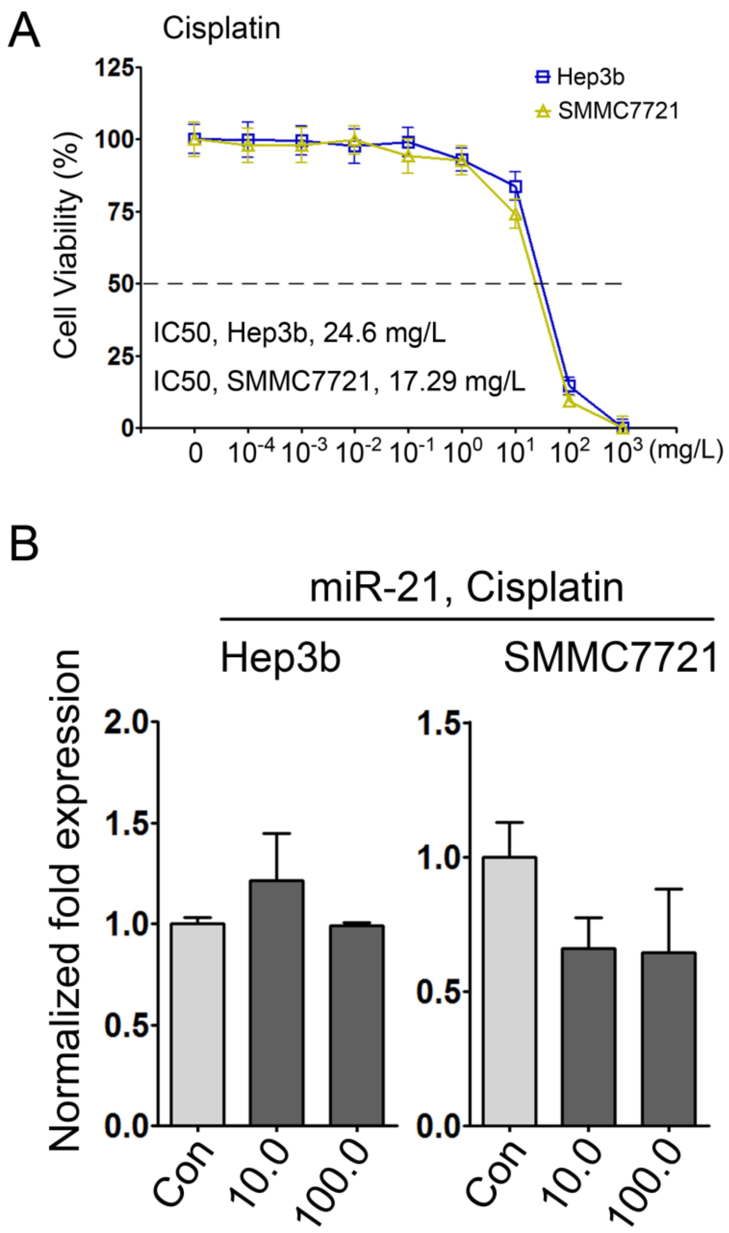

Figure. S7: miR-21 expression is evaluated in HCC cells with cisplatin treatment. A, IC50 value of cisplatin was determined in HCC cells by evaluating cell viability using the CCK-8 assay. **B,** qRT-PCR analysis is conducted to evaluate the expression of miR-21 in Hep3b and SMMC7721 cells with cisplatin treatment. U6 snRNA serves as a control.
